# Development and Internal Validation of a Predictive Model for Adult GH Deficiency Prior to Stimulation Tests

**DOI:** 10.3389/fendo.2021.737947

**Published:** 2021-09-24

**Authors:** Fabio Bioletto, Mirko Parasiliti-Caprino, Alessandro Maria Berton, Nunzia Prencipe, Valeria Cambria, Ezio Ghigo, Silvia Grottoli, Valentina Gasco

**Affiliations:** Division of Endocrinology, Diabetology and Metabolism, Department of Medical Sciences, University of Turin, Turin, Italy

**Keywords:** growth hormone deficiency, hypopituitarism, IGF-I, pre-test probability, predictive model

## Abstract

**Background:**

The diagnosis of adult GH deficiency (GHD) relies on a reduced GH response to provocative tests. Their diagnostic accuracy, however, is not perfect, and a reliable estimation of pre-test GHD probability could be helpful for a better interpretation of their results.

**Methods:**

Eighty patients showing concordant GH response to two provocative tests, i.e. the insulin tolerance test and the GHRH + arginine test, were enrolled. Data on IGF-I values and on the presence/absence of other pituitary deficits were collected and integrated for the estimation of GHD probability prior to stimulation tests.

**Results:**

An independent statistically significant association with the diagnosis of GHD was found both for IGF-I SDS (OR 0.34, 95%-CI 0.18-0.65, p=0.001) and for the presence of other pituitary deficits (OR 6.55, 95%-CI 2.06-20.83, p=0.001). A low (<25%) pre-test GHD probability could be predicted when IGF-I SDS > +0.91 in the presence of other pituitary deficits or IGF-I SDS > -0.52 in the absence of other pituitary deficits. A high (>75%) pre-test GHD probability could be predicted when IGF-I SDS < -0.82 in the presence of other pituitary deficits or IGF-I SDS < -2.26 in the absence of other pituitary deficits.

**Conclusion:**

This is the first study that proposes a quantitative estimation of GHD probability prior to stimulation tests. Our risk class stratification represents a simple tool that could be adopted for a Bayesian interpretation of stimulation test results, selecting patients who may benefit from a second stimulation test and possibly reducing the risk of wrong GHD diagnosis.

## Introduction

Adult GH deficiency (GHD) is a heterogeneous disorder that may result from a variety of causes, including structural lesions, genetic abnormalities, traumas, infiltrative diseases, surgery or irradiation to the pituitary gland and/or hypothalamus (1–5). This condition is characterized by altered body composition, glucose intolerance, abnormal lipid profiles, premature atherosclerosis, osteoporosis, impaired quality of life, and increased mortality (6–9). GH replacement treatment improves most of these abnormalities (10–14); however, due to the high cost of GH therapy (14, 15) and to the concerns of potential long-term risks, particularly the development of diabetes mellitus or malignancies, it is crucial to establish a correct diagnosis in order to offer GH replacement to truly GH-deficient adults (1, 12, 14).

Adult GHD diagnosis depends on the demonstration of a subnormal rise in peak serum GH level in response to one or more GH stimulation tests (1, 16–18). The most relevant stimulation tests to this scope are the insulin tolerance test (ITT), the GH-releasing-hormone + arginine (GHRH + ARG) test, the GHRH + GH-releasing-peptide-6 (GHRH + GHRP-6) test, and the glucagon test (1, 16–18). Their accuracy for GHD diagnosis, though high, is not perfect. Their estimated sensitivity ranges from 87% to 96%, while their estimated specificity from 79% to 92%, depending on the considered study (19–22). This can make the recognition of GHD challenging, and may possibly result in a wrong diagnosis. This picture is further complicated by the interaction between GHD and body mass index (BMI); in fact, obesity is a state of functional relative reduction of GH production, both in terms of spontaneous secretion (23–25) and in terms of responsiveness to stimulation tests (26, 27). To mitigate this issue, specific BMI-related cut-offs have been proposed for most GH provocative tests (20–22), including lately the ITT (28), but false-negative and false-positive results may still occur.

In light of this, the results of a stimulation test should not be interpreted as an unquestionable dichotomous answer on patient’s diagnosis. On the contrary, it should be encouraged to think to the process of GHD diagnosis through a Bayesian approach, in which any additional test result modifies (upward or downward) the probability that a given patient has GHD, as already discussed and suggested for many other medical and endocrinological conditions (29–33). This Bayesian approach to the GHD diagnostic work-up is methodologically well-grounded and allows a more efficient handling and interpretation of stimulation test results, but poses a great problem, i.e. a reliable quantitative estimate of pre-test GHD probability based on other presenting features.

As reported in literature and in current guidelines (1, 34–36), the two most important parameters predicting a final diagnosis of GHD are the presence/absence of other pituitary deficits and the IGF-I values, considered in terms of age-specific standard deviation score (SDS). The information that can be deduced from these features might thus be seen as a proxy for the estimation of GHD probability prior to stimulation tests. Up to date, however, there is no shared, accurate and standardized model that quantitatively estimates this risk. The aim of this study was to develop and internally validate such a model.

## Methods

### Patient Selection

Data of all patients with a history of pituitary disease who underwent two different stimulation tests for the evaluation of GH secretion at the Neuroendocrinology Clinic of our Center between January 2017 and January 2019 were collected from prospective registries and analyzed retrospectively.

The two stimulation tests used for the dynamic evaluation of GH response were the ITT and the GHRH + ARG test. ITT was performed by intravenous injection of 0.1-0.15 IU/kg of regular insulin at 0 min in normal weight and overweight/obese subjects, respectively; blood sampling for GH was performed every 15 min from 0 to +90 min; adequate hypoglycemia was defined as the achievement of a glucose level < 40 mg/dl. GHRH + ARG test was performed by intravenous injection of 1 µg/kg of GHRH at 0 min and of 0.5 g/kg of ARG from 0 to +30 min; blood sampling for GH was performed every 15 min from +30 to +60 min. All subjects underwent the two testing sessions in random order and at least 3 days apart. In the case of patients with functional pituitary adenomas, all tests for the assessment of GH deficiency were conducted after achieving the cure of the underlying pathology. Similarly, no tests were performed in patients presenting other potential interferents with the functional evaluation of GH/IGF-I axis (e.g., catabolic states, liver cirrhosis, end-stage chronic kidney disease, altered and uncorrected thyroid function, exogenous glucocorticoid treatment other than replacement therapy).

Approval from local ethics committees was obtained for the analysis of patient data. Written informed consent was obtained from all included patients.

### Data Collection

For each patient, all the following data were collected: gender, age, BMI, IGF-I values, presence/absence of other pituitary deficiencies apart from GHD, peak GH value at ITT, peak GH value at GHRH + ARG test. The diagnosis of other pituitary deficiencies was made according to current international guidelines (37). The GH response to GHRH + ARG test was defined as normal when GH > 11.0 μg/l for lean subjects, when GH > 8.0 μg/l for overweight subjects and when GH > 4.0 μg/l for obese subjects (1, 21). The GH response to ITT was defined as normal when GH > 3.5 μg/l for lean subjects and when GH > 1.3 μg/l for overweight or obese subjects, in agreement with a recent paper that has identified these as the best BMI-related GH cut-offs for the diagnosis of GHD at ITT (28). Patients with a concordantly normal response to both tests were considered as having a normal function of the somatotroph axis. Patients with concordantly deficient response to both tests were considered as being affected by GHD.

### Analytical Methods

Serum GH levels (μg/l) were measured in duplicate by IRMA method (IRMA GH, Beckman Coulter, Czech Republic). The sensitivity of the assay was 0.033 µg/l. The inter- and intra-assay coefficients of variation (CV) were 9.0-14.0% and 2.4-6.5%, respectively. Serum IGF-I levels (μg/l) were measured in duplicate by RIA method (SM-C-RIA-CT, DIAsource ImmunoAssays, Belgium) after acid-ethanol extraction to avoid interference by binding proteins. The sensitivity of the method was 0.25 μg/l. The inter- and intra-assay CV were 6.8-14.9% and 4.5-7.0%, respectively. IGF-I levels are expressed both as an absolute value and as SDS. The SDS was calculated for each subject, in accordance with the published normality data on a population of 547 healthy Italian subjects (38), as the difference between patient’s IGF-I value and age-specific mean IGF-I value, divided by age-specific IGF-I standard deviation. All other biochemical variables were assayed in plasma or serum using standard methods.

### Statistical Analysis

Baseline characteristics of all patients included in the analysis are summarized using mean and standard deviation for continuous variables and percent values for categorical data. Between-group differences were evaluated by Student t-test for continuous variables, and by either chi-squared test or Fisher’s exact test for categorical variables, as appropriate.

A multivariate logistic regression model was used to examine the predictive performance of IGF-I SDS and presence/absence of other pituitary deficits for the final diagnosis of GHD. In order to reduce the potential bias deriving from class imbalance, undersampling of the majority class was adopted to achieve a 1:1 ratio between patients with and without GHD (39). Randomness in estimates was reduced by iterating the undersampling process ten times and averaging the retrieved coefficients. Model calibration was evaluated at each iteration by the Hosmer-Lemeshow test. Final model performance was evaluated on the original dataset by the area under curve (AUC) at Receiver Operating Characteristic (ROC) analysis. A ten-fold cross-validation algorithm was adopted for internal validation, in order to provide an estimate of model performance on unseen data. After a random split of the original sample into ten groups, the described modeling process was entirely repeated in nine of them, and its performance was evaluated in the tenth. The process was then repeated ten times, rotating the validation group at each round. Final model performance was obtained as the average performance over the ten iterations.

A p-value < 0.05 was adopted for the definition of statistical significance. Statistical analysis was performed using STATA 16 (StataCorp, College Station, Texas, USA).

## Results

### General Characteristics of the Study Population

One hundred and twenty-three patients underwent both ITT and GHRH + ARG test in our Center between January 2017 and January 2019, in the appropriate clinical context for the suspicion of GHD according to the international guidelines (1). Of these, 3 patients were excluded because of the lack of informed consent, and 14 patients were excluded due to the non-achievement of adequate hypoglycemia (glucose < 40 mg/dl) during ITT. Among the remaining 106 patients, 26 were excluded due to discordant results between ITT and GHRH + ARG test. Therefore, finally, 80 patients were included in our final analysis. In 24 of these (30.0%), both stimulation tests concordantly showed a normal GH response; these patients were thus considered as having a normally functioning somatotroph axis. In the remaining 56 (70.0%), both stimulation tests concordantly showed a deficient GH response; these patients were thus considered as having GHD.

Patients with GHD were slightly older than those without GHD (49.9 ± 11.8 *vs* 43.3 ± 14.8 years, p = 0.032); no difference between the two groups could be noted in terms of gender (37.0% *vs* 50.0% females, p = 0.270). BMI was similar in the two groups, both when expressed as a continuous measure (26.7 ± 5.2 vs 25.1 ± 5.8 kg/m^2^, p = 0.216) and when expressed in terms of weight category (p = 0.807). Other pituitary deficits were more common in patients with GHD than in those without GHD (72.2% *vs* 30.8%, p<0.001). IGF-I levels were lower in patients with GHD than in those without GHD, both when expressed in terms of absolute values (90.0 ± 43.7 *vs* 176.6 ± 73.2 µg/l, p<0.001) and in terms of SDS (-1.03 ± 0.90 *vs* -0.16 ± 0.89, p<0.001). No significant differences between the two groups were found in terms of pituitary pathology (p = 0.272). Clinical characteristics of these two groups are summarized in **Table 1**.

**Table 1:** Baseline clinical characteristics in patients diagnosed with and without GHD.

Variables/parameters	No GHD (n = 26)	GHD (n = 54)	p-value
Age (years); mean ± SD	43.3 ± 14.8	49.9 ± 11.8	0.032
Female sex; n (%)	13 (50.0)	20 (37.0)	0.270
IGF-I (μg/l); mean ± SD	176.6 ± 73.2	90.0 ± 43.7	<0.001
IGF-I SDS; mean ± SD	-0.16 ± 0.89	-1.03 ± 0.90	<0.001
Presence of other pituitary deficiencies; n (%)	8 (30.8)	39 (72.2)	<0.001
Number of other pituitary deficiencies; n (%)			0.002
None	18 (69.2)	15 (27.8)	
One	5 (19.2)	11 (20.4)	
Two	1 (3.9)	11 (20.4)	
Three or more	2 (7.7)	17 (31.4)	
Prevalence of other specific pituitary deficiencies			
HPA axis deficiency; n (%)	5 (19.3)	29 (53.7)	0.003
HPG axis deficiency; n (%)	4 (15.4)	28 (51.9)	0.002
HPT axis deficiency; n (%)	4 (15.4)	27 (50.0)	0.003
ADH deficiency; n (%)	1 (3.9)	2 (3.7)	0.698^a^
BMI (kg/m^2^); mean ± SD	25.1 ± 5.8	26.7 ± 5.2	0.216
BMI category; n (%)			0.807
Normal (BMI < 25 kg/m^2^)	12 (46.2)	21 (38.9)	
Overweight (BMI ≥ 25 kg/m^2^ and < 30 kg/m^2^)	8 (30.8)	20 (37.0)	
Obese (BMI ≥ 30 kg/m^2^)	6 (23.1)	13 (24.1)	
Pituitary disease; n (%)			0.272
Sellar mass	22 (84.6)	37 (68.5)	
Empty sella (primary or secondary)	1 (3.8)	7 (13.0)	
Other (Pituitary hypoplasia, Idiopathic hypopituitarism, Traumatic brain injury)	3 (11.6)	10 (18.5)	
Type of sellar mass; n (%)^b^			0.253^a^
Non-functioning adenoma	14 (63.7)	24 (64.9)	
PRL-secreting adenoma	6 (27.3)	6 (16.2)	
GH-secreting adenoma	0 (0.0)	2 (5.4)	
ACTH-secreting adenoma	1 (4.5)	0 (0.0)	
TSH-secreting adenoma	1 (4.5)	0 (0.0)	
Craniopharyngioma	0 (0.0)	3 (8.1)	
Rathke’s cleft cyst	0 (0.0)	2 (5.4)	

a p-value at Fisher’s exact test.

b Percentages calculated with respect to the total number of patients presenting a sellar mass (i.e., 22 patients among those without GHD and 37 patients among those with GHD).

ACTH, adrenocorticotropic hormone; ADH, anti-diuretic hormone; BMI, body mass index; GH, growth hormone; GHD, growth hormone deficiency; HPA, hypothalamus-pituitary-adrenal; HPG, hypothalamus-pituitary-gonads; HPT, hypothalamus-pituitary-thyroid; IGF-I, insulin-like growth factor I; PRL, prolactin; SD, standard deviation; SDS, standard deviation score; TSH, thyroid-stimulating hormone.

### Model Construction and Internal Validation

A clinical prediction model for the diagnosis of GHD was constructed by multivariate logistic regression. The variables included in the model, chosen according to data from the existing literature, were IGF-I SDS and the presence/absence of other pituitary deficits. An independent statistically significant association with the diagnosis of GHD was found both for IGF-I SDS (OR 0.34, 95%-CI 0.18-0.65, p=0.001) and for the presence of other pituitary deficits (OR 6.55, 95%-CI 2.06-20.83, p=0.001) (**Table 2**).

**Table 2:** GHD prediction by multivariate logistic regression on the full dataset.

Predictor	OR	95% CI	p-value
IGF-I SDS	0.34	0.18-0.65	0.001
Presence of other pituitary deficiencies	6.55	2.06-20.83	0.001

CI, confidence interval; GHD, growth hormone deficiency; IGF-I, insulin-like growth factor I; OR, odds ratio; SDS, standard deviation score.

In order to reduce class imbalance, a random undersampling of the majority class was performed and iterated ten-times. Final model parameters were retrieved through averaging of regression coefficients over these ten iterations. This allowed the calculation of the probability (P) of GHD according to the following formula: P = e^z^/(1 + e^z^), where z = 1.82 * other pituitary deficits (yes=1, no=0) – 1.27 * IGF-I SDS – 1.77. The Hosmer-Lemeshow test did not reveal any significant miscalibration (p > 0.30 in all iterations).

**Figure 1:**
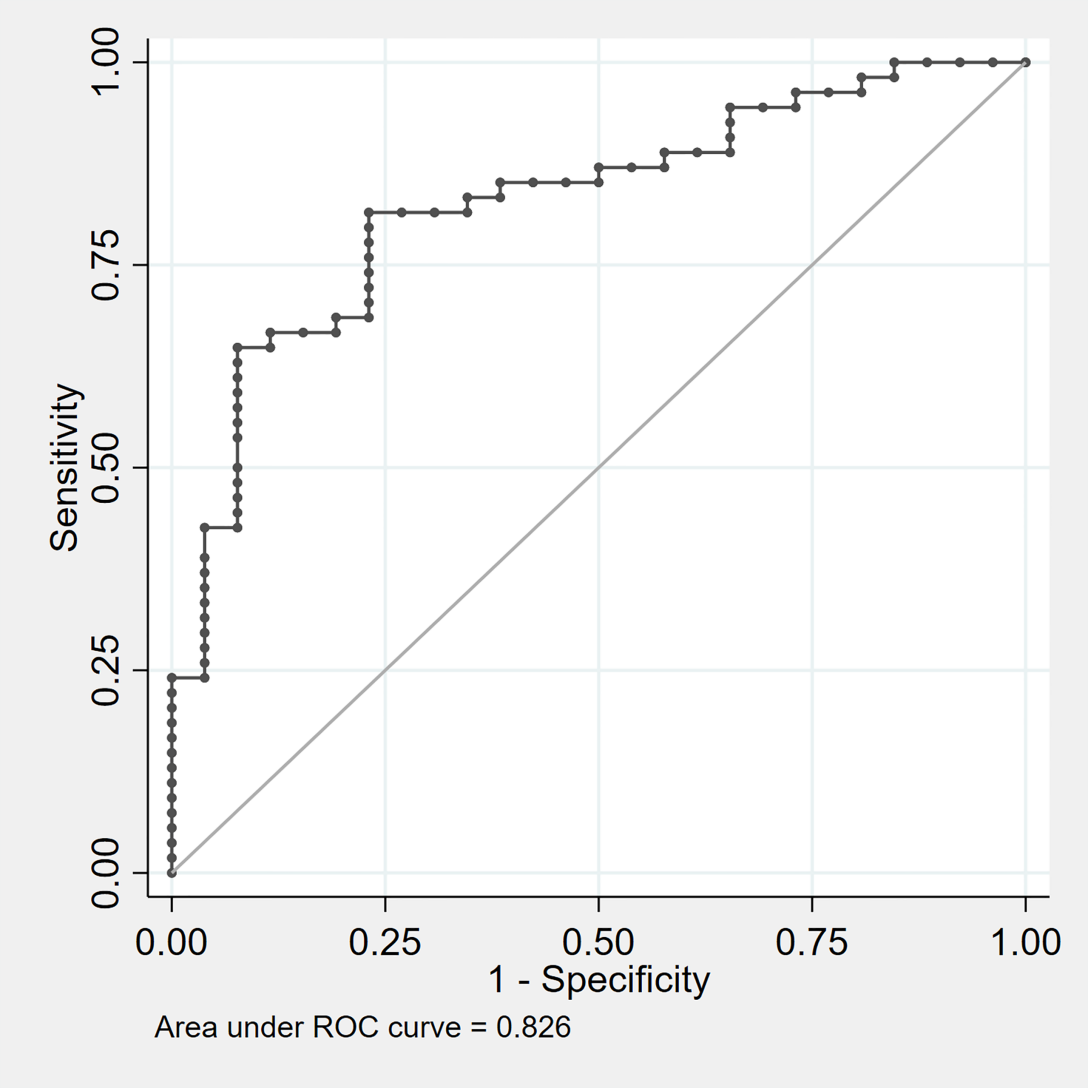
ROC curve evaluating the diagnostic performance of the composite model predictor z = 1.82 * other pituitary deficits (yes =1 , no = 0) – 1.27 * IGF-I SDS – 1.77. ROC, receiver operating characteristic.

The predictive performance of this model was assessed on the original dataset by the calculation of the AUC at ROC analysis, which was equal to 0.826 (**Figure 1**). Notably, the model performance was comparable in lean subjects (AUC 0.849) and in overweight/obese subjects (AUC 0.816). Internal validation of the model was performed through ten-fold cross-validation, as already described. The final estimation of the model performance on unseen data, obtained as the average AUC over the ten iterations, was equal to 0.820, thus reassuring about a substantially null overfitting effect.

### Risk Class Stratification

In order to simplify the use of the model in clinical practice, the equation of the logistic regression model was used to retrieve GHD probabilities estimated according to the considered predictive variables. Results were stratified according to the presence or the absence of other pituitary deficits and for each case the cut-offs of IGF-I SDS predicting a probability of GHD of 25%, 50% and 75% were retrieved. In the absence of other pituitary deficits, these cut-offs were -0.52, -1.39 and -2.26, respectively. In the presence of other pituitary deficits, these cut-offs were +0.91, +0.04 and -0.82, respectively (**Table 3**).

**Table 3:** IGF-I SDS values predicting a pre-test GHD probability of 25%, 50% and 75%, stratified according to the presence or the absence of other pituitary deficits.

IGF-I SDS in the absence of other pituitary deficits	IGF-I SDS in the presence of other pituitary deficits	Pre-test GHD probability
-0.52	+0.91	25%
-1.39	+0.04	50%
-2.26	-0.82	75%

GHD, growth hormone deficiency; IGF-I, insulin-like growth factor I; SDS, standard deviation score.

## Discussion

In this study, we developed and internally validated a multivariate prediction model for the estimation of GHD probability prior to stimulation tests. Our model showed a good predictive power for the discrimination between subjects with and without a final GHD diagnosis, with an AUC of 0.826. To facilitate its clinical use, we also retrieved specific cut-offs corresponding to GHD probability of 25%, 50% and 75%.

As previously said, GH stimulation tests are not perfect in sensitivity and specificity (19–22), and their interpretation should not therefore be viewed as a dichotomous response on patient’s diagnosis. Pre-test probability estimation is of key importance to allow a proper interpretation of the test results. The presence of other pituitary deficits and the IGF-I values, evaluated in terms of age-dependent SDS, have been widely recognized as predictors of GHD by many authors (34–36), as well as by current Endocrine Society guidelines (1). These results were confirmed also in our cohort, and were used to quantitatively develop a multivariate model for the estimation of GHD probability prior to stimulation tests.

The overall accuracy of the model was not sufficient to discriminate by itself between patients with and without GHD, but this was neither an expected nor a desired result. In fact, IGF-I levels depend on several factors other than GH stimulation, such as nutritional status (40, 41) and genetic polymorphisms (42); concordantly, even if the presence/absence of other pituitary deficits can be seen as a rough approximation of the functional integrity of pituitary gland, the specific functional status of each axis is ultimately independent from that of the others.

Therefore, a provocative test should still represent a mandatory step for GHD diagnosis in most cases; our model, however, could be of significant help in clinical practice for the identification of false-positive and/or false-negative stimulation test results. In fact, by Bayes theorem (29–31), when applying a diagnostic test characterized by approximately 90% sensitivity and specificity, if the pre-test probability of a disease or condition is < 25%, the post-test probability still remains < 75% after a positive test result. Conversely, if the pre-test probability is > 75%, the post-test probability still remains > 25% after a negative test result. Therefore, in patients with a low pre-test probability of GHD, a blunted GH response to one stimulation test should be interpreted with caution, and an indication for further testing should be considered; the same applies with reversed parts, i.e. in patients with a high pre-test probability of GHD and a normal GH response to one stimulation test.

**Figure 2:**
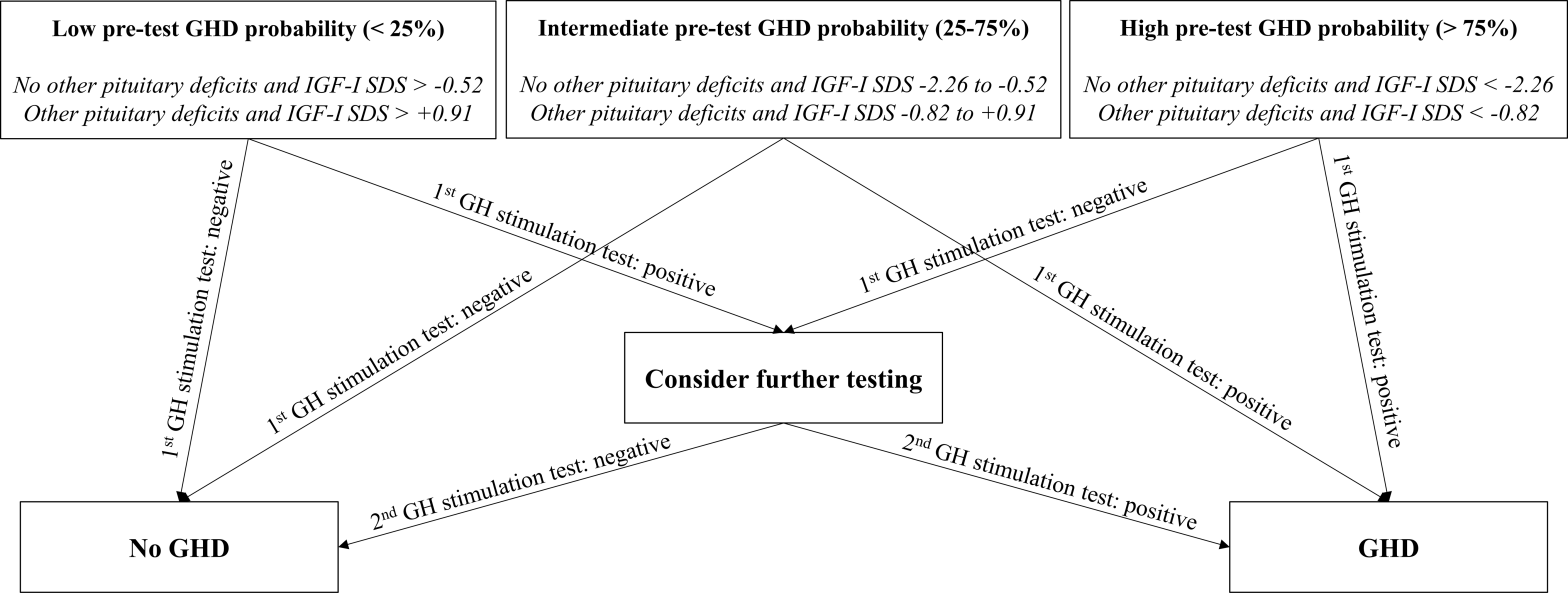
Flow-chart proposal for integrated GHD diagnosis. GH, growth hormone; GHD, growth hormone deficiency; IGF-I, insulin-like growth factor I; SDS, standard deviation score.

We have undertaken this study with the specific aim of improving the diagnosis of adult GHD, which is currently based solely on the failure of GH response to one stimulation test. However, as already mentioned, even in the appropriate clinical context, each stimulation test has some limitations, with the possibility of false-positive and/or false-negative results. To date, the interpretation of the response to any stimulation test is based on cut-offs that do not take into account the higher or lower pre-test probability of GHD, thus ignoring a whole part of complementary information which could better refine the diagnostic process. In **Figure 2**, therefore, we propose a flow-chart for a comprehensive GHD diagnostic work-up, integrating both pre-test GHD probability estimation and stimulation test results. In line with what has just been discussed, this flow-chart suggests a specific attention in patients in which stimulation test results appear to be discordant with respect to the estimated pre-test GHD probability. In these cases, a second stimulation test may be advisable for a safer confirmation or exclusion of GHD diagnosis.

The main strength of our study was the use of two recognized and concordant GH stimulation tests as the reference standard for the definition of GH deficiency/sufficiency, which gave a strong support to the reliability of GHD diagnosis. Moreover, the internal validation of our model conferred higher statistical consistency to the obtained results.

Our study had also some limitations. The first one was related to its retrospective design; however, the retrieved data were prospectively collected and, most notably, the recall of baseline clinical features for each patient was based only on data retrieved from clinical reports preceding the beginning of any biochemical work-up by stimulation tests. A second limitation might be related to the high prevalence of patients with GHD in the study population, which is likely a consequence of the tertiary nature of our Center; in order to mitigate the effect of class imbalance, parameter tuning was performed on balanced subsets through random undersampling of the majority class; nevertheless, the proposed model should be applied with caution in patient populations in which the prevalence of GHD differs markedly from 50%.

In conclusion, this is the first study that proposes a quantitative estimation of GHD probability prior to stimulation tests. Our final flow-chart represents a simple tool that could be adopted for a Bayesian interpretation of stimulation test results, selecting patients who may benefit from a second stimulation test. The proposed approach could make a significant contribution towards the standardization of GHD diagnostic process, and would likely result in a reduction of the risk of wrong GHD diagnoses.

## Data Availability Statement

The raw data supporting the conclusions of this article will be made available by the authors upon request, without undue reservation.

## Ethics Statement

The studies involving human participants were reviewed and approved by Comitato Etico Interaziendale A.O.U. Città della Salute e della Scienza di Torino—A.O. Ordine Mauriziano—A.S.L. Città di Torino. The patients/participants provided their written informed consent to participate in this study.

## Author Contributions

FB conceptualized the work, performed the data analysis, and contributed to the manuscript writing. VC contributed to the data collection and manuscript writing. MP-C, AMB, and NP contributed to data interpretation and manuscript writing. SG and EG supervised the manuscript drafting. VG contributed to work conceptualization, data analysis, manuscript writing, and final draft supervision. All authors contributed to the article and approved the submitted version.

## Conflict of Interest

The authors declare that the research was conducted in the absence of any commercial or financial relationships that could be construed as a potential conflict of interest.

## Publisher’s Note

All claims expressed in this article are solely those of the authors and do not necessarily represent those of their affiliated organizations, or those of the publisher, the editors and the reviewers. Any product that may be evaluated in this article, or claim that may be made by its manufacturer, is not guaranteed or endorsed by the publisher.
